# The Effects of COVID-19 on Physicians’ Perceived Ability to Provide Care for Patients With Type II Diabetes Mellitus

**DOI:** 10.7759/cureus.29135

**Published:** 2022-09-13

**Authors:** Abbas Abidi, Francis Demiraj, Garry Berdichevskiy, Krisha Gupta, Daniel Epstein, Shawn Kurian, Antony Aranyos, Avidor Gerstenfeld, Nasser Assadi, Chulou H Penales

**Affiliations:** 1 Dr. Kiran C. Patel College of Osteopathic Medicine, Nova Southeastern University, Davie, USA; 2 Critical Care, Westside Regional Medical Center, Plantation, USA

**Keywords:** physician satisfaction, covid 19 impact of lockdown, drug compliance, telehealth appointments, diabetes type 2

## Abstract

Background and objective

The coronavirus disease 2019 (COVID-19) pandemic, caused by severe acute respiratory syndrome coronavirus 2 (SARS-CoV-2), presents multiple, diverse challenges to providing appropriate medical care, especially in terms of medication and treatment adherence for chronic diseases such as type 2 diabetes mellitus (T2DM). The COVID-19 pandemic has exacerbated these barriers by potentially forcing physicians to modify their treatment plans due to limitations on in-person visits and changes to patients' financial and social support systems. It remains uncertain whether physicians believe they can provide the same standard of care using telehealth technology or other means to their patients during the pandemic. The goal of this study was to explore physician perceptions about their ability to provide care to patients with T2DM during the COVID-19 pandemic.

Methodology

This cross-sectional study collected data between January 25, 2021, and February 2, 2021, using an anonymous, self-administered online survey involving DO and MD physicians including residents treating patients with T2DM. The survey was administered via REDCap and collected data on participant demographics, attitudes, perceptions, knowledge, and prior and current (COVID-19-era) experience with care for T2DM patients. Physicians registered with the Florida Department of Health with publicly available emails were invited to participate.

Results

The survey showed that during the COVID-19 pandemic, 57.9% of physicians (n=48) believed that their patients have a weaker social support system; 68.7% (n=57) modified their patient care plans due to patients' financial difficulties; 78.4% (n=65) believed a regular physical exam is necessary to properly treat patients; 48.2% (n=40) did not believe they had a more complete picture of the case with remote consultations; 47.0% (n=39) were not as satisfied with remote consultations as with face-to-face patient visits; 68.7% (n=57) believed telehealth is necessary to adequately treat patients; 38.5% (n=32) have been less likely to refer their patients to other providers or specialists; 45.8% (n=38) reported concerns over admitting their patients to the hospital for acute medical care; 61.5% (n=51) reported having more patients delay scheduling their routine follow-up care; 61.5% (n=51) believed their patients have been less compliant with the healthcare plans recommended to them.

Conclusions

The study showed that COVID-19 has significantly impacted physicians’ perceptions and abilities to provide care for patients with T2DM. COVID-19 has negatively impacted several crucial aspects of diabetes management, including consistent in-person examinations, social support, and referral to other required services, which could result in long-term consequences for these patients. Furthermore, our study suggests that physicians may not be as satisfied with the care they are able to provide via remote consultations as they are with in-person visits, which has significant implications as we move toward a more telehealth-driven healthcare delivery system.

## Introduction

The coronavirus disease 2019 (COVID-19) pandemic, caused by severe acute respiratory syndrome coronavirus 2 (SARS-CoV-2), is associated with multiple, diverse challenges to providing appropriate medical care, especially in terms of medication and treatment adherence to chronic diseases, such as type 2 diabetes mellitus (T2DM). Pre-existing comorbidities, such as DM, seem to increase the risk of serious SARS-CoV-2 infection and lead to increased disease severity and mortality [1]. As of 2017, 24.6 million Americans suffer from T2DM, with its economic effects estimated in the billions [2]. In addition, the pandemic itself poses challenges that affect healthcare systems as a whole, as outbreaks in areas may lead to the deployment of resources away from patients with chronic illnesses [3]. Thus, providers might rely on self-adherence treatment protocols, which include supplies of medications that might need to last several months, which can be complicated by socioeconomic factors, including job and health insurance loss [4]. The sensitivity of the treatment approach to patients with T2DM was also complicated by the higher risk of severe disease [5]. The possibility of severe disease drove a general trend toward the increase in the use of telehealth visits [6]. Even more specifically, 27% of diabetic patients used telehealth in the second quarter of 2020, the period relevant to this study [7].

The approach to the management of patients with T2DM is complicated by the fact that they often present to clinics with other chronic conditions, and also present to physician’s offices more often [8,9]. The associated complications pose serious questions as to how to approach the management of patients with T2DM in the event of disasters in general. During disaster events, though “medication refill is an immediate health need, making the prescription of medications for pre-existing conditions an increasing burden of medical relief activities at a time when acute needs are also over-whelming,” [10] a pandemic is different in that the scale of the disaster is larger and by necessity may include many just-in-time logistical chains that could potentially be affected [11-13].

For a complex chronic disease such as diabetes, a crucial part of long-term management is the level of social support among patients. The most immediate components of social support include a patient’s close social network of family and caregivers with self-esteem and worthiness being other close factors. Higher levels of social support have been shown to improve patient self-efficacy and medication adherence, which ultimately improves glycemic control [14]. With a key aspect of the lockdown policy being social isolation, it was imperative that our study examined physician perception of patient social support. With 57.9% of our surveyed physicians finding a weaker support system among their patients, a key aspect of diabetes management was shown to be negatively affected by the impact of the COVID-19 lockdown and telehealth implementation. Our finding of perceived decreased social support is in line with other published findings such as those of an Austrian study that also found that this effect was more pronounced in individuals with vulnerabilities such as depression and anxiety [15]. A key implication of this trend is that physicians with patient populations that score higher on depression and anxiety scales would be more likely to feel less social support on their patient’s behalf and potentially alter disease management. Social support issues such as self-efficacy and self-blame have been shown to be barriers to insulin initiation, which strengthens the need to further understand how physicians assess their patient’s self-image in a telehealth encounter and how to improve the patient's social standing in a socially isolated environment [16].

Thus, given the realities of the pandemic lockdown, caregivers must first be convinced that telehealth is a viable option for the management of their patients, and their views as to how the medium affects patient-physician interaction are vital. The ultimate aim of this paper is to elucidate how physicians view the medium and find areas that are ripe for further research.

## Materials and methods

Our cross-sectional study collected data between January 25, 2021, and February 2, 2021, from physicians and residents treating patients with T2DM, by using an anonymous, self-administered online survey. The Nova Southeastern University Institutional Review Board reviewed and approved this study (IRB number: 2020-661). The inclusion criteria for the study was an active physician medical license in the state of Florida registered under the Florida Department of Health (FDOH). We primarily targeted family medicine, geriatrics, internal medicine, and endocrinology as our specialties of interest as we wanted to focus on physicians who tended to see higher volumes of T2DM patients. Physicians who met this criterion were invited to participate via their email registered with the FDOH. These physician data were compiled from publicly available text files on the FDOH website and imported into an Access database, where they were combined using a combo primary key, which matched the physician data to their email registered with the FDOH. The data were further subdivided into internal medicine physicians, family medicine physicians, and endocrinologists, who were emailed a REDCap survey link. Survey questions and statements were created by our group under the guidance of our research advisor, Dr. Robin Jacobs. No pre-testing was done as we did not want to confound the results given the evolving nature of the COVID-19 lockdown policy. The survey was constructed and operated on REDCap, a browser-based software that securely collects anonymous participant responses. The survey assessed the demographic information of each participant by allowing physicians to input their age, race, and gender, The survey posed statements to participants on topics such as attitudes, perceptions, knowledge, and prior and current (COVID-19-era) experience regarding care for T2DM patients. Survey participants would then respond whether they strongly agreed, agreed, somewhat agreed, neither agreed nor disagreed, somewhat disagreed, disagreed, and strongly disagreed with the posed statement. Statistical analyses were conducted by using SPSS Statistics v.26 (IBM Corp., Armonk, NY), which allowed us to further group survey responses as general agreement or disagreement when appropriate.

## Results

A total of 83 physicians (26 females, 55 males, and two non-binary individuals) participated in this research after having the study explained to them and then signing an Institutional Review Board-approved consent form. The mean age for the entire group was 56 ± 10.5 years (range: 31-85 years). Participant entry requirements were as follows: being a physician or resident physician practicing in the United States and treating patients with T2DM. The specialties of these physicians were family medicine (n=47), internal medicine (n=21), endocrinology (n=1), geriatrics (n=6), and other (n=8).

Race and ethnicity were self-reported by study participants and race categories used were based on the Revisions to the Standards for the Classification of Federal Data on Race and Ethnicity [17]. The participant self-reported data on their race/ethnicity were as follows: White/Caucasian (n=66), Black/African American (n=2), Asian (n=7), American Indian/Alaska Native (n=1), multiracial/biracial (n=4), other (n=1); two participants preferred not to answer this question. A few participants also self-reported as Hispanic/Latinx (n=10).

**Figure 1:**
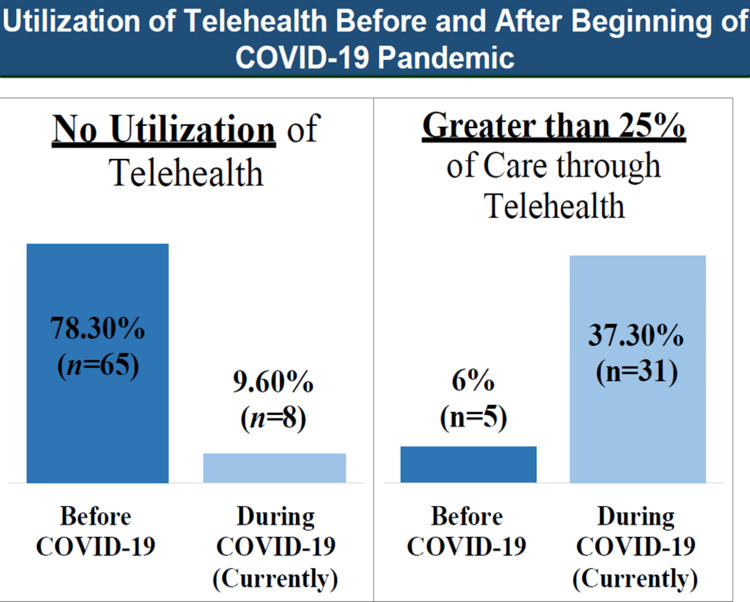
Utilization of telehealth before and during the pandemic COVID-19: coronavirus disease 2019

Figure 1 depicts the expected surge in the utilization of telehealth from before the COVID-19 pandemic to the evaluation period during the lockdown, with a 31.3% increase in physicians reporting that at least a quarter of their care was being provided through telehealth. 

The survey showed that during the COVID-19 pandemic, 57.9% of the 83 physicians surveyed (n=48) believed that their patients currently have a weaker social support system; 68.7% (n=57) modified their patient care plans due to patients' financial difficulties; 78.4% (n=65) believed a regular physical exam is necessary to properly treat patients; 48.2% (n=40) did not believe they had a more complete picture of the case with remote consultations; 50.7% (n=42) reported difficulty with seeing patients in person due to the COVID-19 lockdown policy; 47.0% (n=39) were not as satisfied with remote consultations as with face-to-face patient visits; 68.7% (n=57) believed telehealth is necessary to adequately treat patients; 38.5% (n=32) have been less likely to refer their patients to other providers or specialists; 45.8% (n=38) reported concern over admitting their patients to the hospital for acute medical care; 61.5% (n=51) reported having more patients delay scheduling their routine follow-up care; and 61.5% (n=51) believed that their patients have been less compliant with the healthcare plans recommended to them. Tables 1-3 show physician responses both as a frequency and percentage of total responses to our posed statements on COVID-19-related issues. 

**Table 1:** Physician survey responses to posed statements, n (%)

	Strongly disagree	Disagree	Somewhat disagree	Neither agree nor disagree	Somewhat agree	Agree	Strongly agree	N/A
I believe my patients have a weaker social support system	4 (4.8%)	10 (12.0%)	5 (6.0%)	14 (16.9%)	14 (16.9%)	22 (26.5%)	12 (14.5%)	2 (2.4%)
I have modified my patient care plan due to restrictions in following up with my patients	13 (15.7%)	10 (12.0%)	7 (8.4%)	9 (10.8%)	13 (15.7%)	17 (20.5%)	9 (10.8%)	5 (6.0%)
I believe that a regular physical exam is necessary to properly treat patients with type II diabetes mellitus	4 (4.8%)	2 (2.4%)	8 (9.6%)	4 (4.8%)	11 (13.3%)	22 (26.5%)	32 (38.6%)	0 (0.0%)
I can have a more complete picture of the case with remote consultations	9 (10.8%)	19 (22.9%)	12 (14.5%)	11 (13.3%)	9 (10.8%)	15 (18.1%)	5 (6.0%)	3 (3.6%)
I have had difficulty seeing patients in person due to COVID-19-related policies	7 (8.4%)	13 (15.7%)	9 (10.8%)	8 (9.6%)	14 (16.9%)	13 (15.7%)	15 (18.1%)	4 (4.8%)
I have been less likely to refer my patients to other providers or specialists	12 (14.5%)	21 (25.3%)	8 (9.6%)	8 (9.6%)	10 (12.0%)	14 (16.9%)	8 (9.6%)	2 (2.4%)
I have more patients delay scheduling their routine follow-up care	3 (3.6%)	8 (9.6%)	9 (10.8%)	7 (8.4%)	13 (15.7%)	21 (25.3%)	17 (20.5%)	5 (6.0%)

**Table 2:** Physician survey responses to posed statements, n (%)

	Strongly disagree	Disagree	Somewhat disagree	Neither agree nor disagree	Somewhat agree	Agree	Strongly agree	N/A
I believe my patients have been less compliant with the healthcare plans that I recommend to them	5 (6.0%)	10 (12.0%)	4 (4.8%)	10 (12.0%)	17 (20.5%)	18 (21.7%)	16 (19.3%)	3 (3.6%)
I have asked my patients about their personal lives (such as social support, mental health, financial issues, or isolation)	5 (6.0%)	0 (0.0%)	2 (2.4%)	1 (1.2%)	6 (7.2%)	29 (34.9%)	38 (45.8%)	2 (2.4%)
I have been able to have adequate visits with my patients (face-to-face or telehealth visits)	6 (7.2%)	5 (6.0%)	4 (4.8%)	1 (1.2%)	11 (13.3%)	23 (27.7%)	29 (34.9%)	4 (4.8%)
I have been able to physically examine my patients	8 (9.6%)	9 (10.8%)	7 (8.4%)	3 (3.6%)	14 (16.9%)	21 (25.3%)	19 (22.9%)	2 (2.4%)
I have had difficulty caring for my patients because I have not seen them in my office	9 (10.8%)	16 (19.3%)	12 (14.5%)	7 (8.4%)	17 (20.5%)	11 (13.3%)	6 (7.2%)	5 (6.0%)
I believe that telehealth is necessary to adequately treat patients with type II diabetes mellitus	3 (3.6%)	5 (6.0%)	4 (4.8%)	13 (15.7%)	13 (15.7%)	21 (25.3%)	23 (27.7%)	1 (1.2%)
...I have been as satisfied with remote consultations as with face-to-face patient visits	18 (21.7%)	14 (16.9%)	7 (8.4%)	12 (14.5%)	13 (15.7%)	11 (13.3%)	3 (3.6%)	5 (6.0%)

**Table 3:** Physician survey responses to posed statements, n (%)

	Strongly disagree	Disagree	Somewhat disagree	Neither agree nor disagree	Somewhat agree	Agree	Strongly agree	N/A
...I am more likely to see type II diabetes mellitus patients via telehealth than other patients in my practice	13 (15.7%)	19 (22.9%)	9 (10.8%)	15 (18.1%)	9 (10.8%)	6 (7.2%)	6 (7.2%)	6 (7.2%)
...I am concerned about admitting my patients with type II diabetes mellitus into the hospital for acute medical care	10 (12.0%)	17 (20.5%)	1 (1.2%)	9 (10.8%)	15 (18.1%)	10 (12.0%)	13 (15.7%)	8 (9.6%)
...I usually initiate insulin therapy for my patients with type II diabetes mellitus	11 (13.3%)	15 (18.1%)	7 (8.4%)	7 (8.4%)	12 (14.5%)	19 (22.9%)	10 (12.0%)	2 (2.4%)
...I believe that the initiation of insulin therapy is one of the most difficult aspects of managing my patients with type II diabetes mellitus	6 (7.2%)	10 (12.0%)	8 (9.6%)	8 (9.6%)	14 (16.9%)	22 (26.5%)	13 (15.7%)	2 (2.4%)
...I have modified my patient care plans due to their financial difficulties	8 (9.6%)	6 (7.2%)	5 (6.0%)	5 (6.0%)	11 (13.3%)	27 (32.5%)	19 (22.9%)	2 (2.4%)
...I have been able to effectively monitor my patients' HbA1c levels	4 (4.8%)	3 (3.6%)	11 (13.3%)	0 (0.0%)	10 (12.0%)	23 (27.7%)	30 (36.1%)	2 (2.4%)
...I have encouraged my patients to modify their lifestyles (such as diet or exercise)	1 (1.2%)	0 (0.0%)	0 (0.0%)	2 (2.4%)	2 (2.4%)	17 (20.5%)	59 (71.1%)	2 (2.4%)

## Discussion

In the evaluation of physician perception of telehealth during COVID-19, it was found that it was essential to assess physicians' attitudes toward in-person visits versus telehealth visits. We found a concerning finding with regard to telehealth as 47.8% voiced dissatisfaction with virtual visits replacing in-person visits. This negative finding for telehealth contrasts with several national COVID-19 surveys, which found a strong positive physician response to telehealth as a substitute.

One area of focus for our study was the impact of COVID-19 and telehealth on routine medical care, a vital aspect of diabetes management. Of note, 61.5% of physicians surveyed in our study believed in some part that COVID-19 and telehealth had led to a delay in scheduling their routine follow-up care. This is in contrast to a recent nationwide survey, where 69% of physicians in an urban environment believed telehealth improved access to care in their practice during COVID-19 [18].

One common theme across medical management during the COVID-19 pandemic is the concern related to in-patient admittance for chronic conditions. Our study looked into this challenge for physicians, with 45.8% voicing concerns about admitting their patients for acute medical care. An analysis published in the Lancet found that diabetic ketoacidosis admissions increased by 6% overall in T2DM patients and there was a 41% increase among those previously diagnosed during the initial phase of the pandemic, which they defined as March 1 to June 30, 2020 [19]. Further analysis is needed to find out if these increased admissions were due to delayed chronic management or other extenuating factors.

In addition, the fact that the pandemic has led to delay or avoidance of care could have significant consequences [20]. It is not difficult to imagine a scenario in which a patient presents to the clinic at a more advanced stage of disease, which might compromise initial treatment protocols as well as the management of patients who develop chronic conditions unrelated to infectious disease [21,22]. Also, there are sociodemographic considerations, since a retrospective study found a 32% decrease in hospital admissions overall during the initial phase of the pandemic [23]. Self-adherence protocols were also complicated, as evidenced by reports that patients with T2DM saw an increase in carbohydrate intake, a decrease in exercise, decreased self-monitoring of blood glucose (SMBG), and widespread mental stress [24].

Though treatment adherence research is a field of remarkable depth, the pandemic presents new obstacles as well as new opportunities to understand patient adherence and compliance with treatment protocols. It will take many years to understand the full toll of the pandemic on chronic diseases, but foundational research has already taken place. In the study by Maddaloni et al., glucose control was studied through analysis of uploaded patient data from two weeks before the lockdown in Italy and the subsequent two weeks. While this data is valuable, it excluded individuals who were unable to upload data onto online platforms, or who had contact with endocrinologists during the lockdown period [25]. It is this group, in particular, that comprised individuals who did not have access to care or the ability to provide their practitioners with relevant compliance data, which are the objects of interest to this paper. Moreover, the data provided by Maddaloni et al. were patient-centered, which is extremely valuable, but suffered from a low sample size and did not focus on the impact of providers in terms of delivering care [25].

The objective of this study involved an analysis of the two-fold concern with the decreased ability of providers to deliver care and patients to provide appropriate compliance data in order to ensure they are following the recommended care protocols for DM patients during the COVID-19 pandemic. Clinical management suggestions for patients with DM, during this time, include a patient-tailored therapeutic approach, with regular remote glucose monitoring and adherence to medical recommendations [26]. What these management suggestions assume is that there is no serious impact on the ability of providers to ensure their patients are following the recommended guidelines, as lockdowns have affected both patients and providers [27]. While the available data show that there was significantly more patient adherence when “the doctor offered more information, asked fewer questions overall, but more questions about compliance in particular, and was more positive and less negative,” [28] this data is based on assumed face-to-face interactions. Since close contact with providers and diabetes patients may risk COVID-19 transmission, telemedicine consultations, among other measures, were suggested by healthcare authorities [28]. The severity of the pandemic has forced the hand of healthcare authorities in this regard, but the overall effect of telemedicine on compliance outcomes should be explored further [28].

While continuing to study the impact of the pandemic and all its associated effects is of utmost importance, it is also vital to understand how the pandemic has affected providers' ability to care for their patients. If providers feel that measures such as telehealth negatively correlate with their ability to provide patients with what those patients need, as well as perceiving decreased compliance from patients, they may be less likely to continue to employ those measures in the future, impacting the management of diabetes patients directly [27]. As the pandemic has progressed, the positive views of physicians towards the medium have seemingly increased. In one study of radiation oncologists, 71% of responding physicians reported that telemedicine did not cause any difference in their ability to treat cancer appropriately [29].

Compliance issues are further complicated by data that suggest that paper diaries, which are used for chronic illness patients to track adherence, show low levels of compliance, with high levels of faked compliance, especially when compared to electronic diaries, which showed compliance rates of upwards of 90% [30]. Furthermore, high rates of noncompliance in lockdown behavior in young populations may increase the risk of transmission to family members with chronic illness, which would undermine strategies developed by health authorities to prevent transmission in the first place [31]. Additionally, telehealth and compliance strategies that rely on technology are dictated by the medium of exchange, namely, access to appropriate technology in the first place. Given the various socioeconomic factors at play with diabetes as well as COVID-19 lockdowns, compliance and care may be affected by the inability to access technology [32]. In this context, it is of vital importance to develop clinical trials and recommendations for the use of telehealth in patient-physician interactions, so as to ensure physician comfort in the future, as well as to maximize the reach of physicians with their patient populations.

Although our study had several strengths, such as the high number of physicians and specialties surveyed, there are some limitations to our findings. One weakness of our study is that all physicians surveyed were practicing in the state of Florida. Other regions/states in the country may have varying levels of internet connectivity, and telehealth implementation may look very different in those areas. For example, a southern California qualitative study found a more positive response from physicians with regard to telehealth implementation [33]. As a result, nationwide physician perception of these changes cannot be expected to easily correlate with our findings. A key feature of our study was that it was carried out during the lockdown period of the COVID-19 pandemic. It is reasonable to estimate that as the time of implementation increased, better monitoring and outcomes could be observed. A 2021 retrospective review that was conducted several months after our study revealed that patients who received telehealth consultations during the COVID-19 lockdown had better glycemic control and unaffected hospital admissions [34].

## Conclusions

Our analysis of physician perception of the management of T2DM during the COVID-19 lockdown period revealed concerning trends for disease management and telehealth implementation during this time. Physicians expressed significant concerns about referrals to other providers and in-patient admission of their patients during the lockdown period. Similar nationwide analyses have found more encouraging signs for telehealth implementation, but further research needs to be done to control for confounding factors such as patient access to technology and time of telehealth implementation.
